# Stage-Stratified Economic Burden, Quality of Life, and Household Financial Coping Strategies in Diabetic Retinopathy in India: A Prospective Study With Exploratory Insurance Projections

**DOI:** 10.7759/cureus.108144

**Published:** 2026-05-02

**Authors:** Harini Tiruvengadakrishnan, Vaishnavi Ravi, Samarapuri A

**Affiliations:** 1 Ophthalmology, Sri Ramachandra Institute of Higher Education and Research, Chennai, IND

**Keywords:** catastrophic health expenditure, diabetic retinopathy, direct and indirect costs, economic burden, financial coping strategies, health insurance projections, india, mental health, prospective study, vision-related quality of life

## Abstract

Background

Diabetic retinopathy (DR) imposes a substantial economic and psychosocial burden in India, yet stage-specific integrated data remain limited. This study aimed to evaluate the stage-wise economic burden, vision-related quality of life (VRQoL), and household financial coping strategies in patients with DR.

Methods

This cross-sectional observational study, conducted between November 2024 and March 2025, included 350 patients with type 2 diabetes and DR stratified as mild non-proliferative diabetic retinopathy (NPDR) (n=95), moderate NPDR (n=112), severe NPDR (n=78), and proliferative DR (PDR, n=65). Direct and indirect costs were assessed using structured interviews and verified from medical records where available. VRQoL was evaluated using the IND-VFQ-33. Catastrophic health expenditure (CHE) was defined as healthcare spending exceeding 40% of non-food household expenditure. Between-group comparisons used one-way ANOVA with Bonferroni-corrected post-hoc pairwise testing for normally distributed continuous variables, Kruskal-Wallis tests for non-normally distributed variables, and χ² or Fisher’s exact tests for categorical variables. Multiple linear regression, with full assessment of multicollinearity (variance inflation factor, VIF) and inspection of residuals for normality and homoscedasticity, was used to identify independent predictors of VRQoL. Exploratory, stage-weighted insurance projections were derived using a simplified, scenario-based break-even model.

Results

Patients with PDR were older and had longer duration of diabetes compared with mild NPDR (15.7 ± 4.9 vs. 7.2 ± 3.8 years; p<0.001). Mean total annual cost increased more than six-fold from INR 20,000 ± 3,000 (95% CI 19,396-20,604) in mild NPDR to INR 130,000 ± 13,000 (95% CI 126,840-133,160) in PDR (p<0.001), with all between-stage pairwise differences remaining significant after Bonferroni correction. CHE affected 12% of mild NPDR, 28% of moderate NPDR, 65% of severe NPDR, and 78% of PDR patients (p<0.001). VRQoL worsened markedly from mild NPDR to PDR (30 ± 8 vs. 90 ± 10; p<0.001), with parallel increases in depression and anxiety scores. Coping strategies included borrowing (58%), loans (42%), asset sales (28%), and withdrawal of children from education (15%). Total economic burden was strongly correlated with VRQoL (Spearman’s ρ = 0.78, 95% CI 0.73-0.82; p<0.001). Exploratory projections estimated an annual insurance contribution of approximately INR 22,000.

Conclusions

Diabetic retinopathy in India is associated with a steep, stage-dependent escalation in economic burden, catastrophic expenditure, and deterioration in VRQoL, with significant intergenerational consequences. Exploratory insurance modeling illustrates a potential pathway for mitigating financial burden alongside prevention and early detection.

## Introduction

Diabetic retinopathy (DR) is one of the most economically consequential complications of diabetes in India [1]. Beyond its role as a leading cause of visual impairment among working-age adults, DR imposes a substantial national economic burden driven predominantly by productivity losses and vision-related disability [2-4]. It is estimated that, among Indians aged ≥40 years, productivity losses attributable to DR and vision impairment alone amount to approximately INR 47,200 crore annually, far exceeding the direct costs of screening and treatment [5]. Annual nationwide screening of the 55.9 million diabetics aged ≥40 years would cost an estimated INR 4,230 crore, while treating approximately 20% of detected cases would add another INR 287 crore. These figures highlight how preventable productivity losses far exceed direct medical costs [6]. These figures highlight the enormous economic consequences of delayed detection and suboptimal disease control at a population level.

At the patient level, DR imposes escalating direct medical and indirect productivity costs with increasing disease severity. Data from South Indian tertiary-care centres suggest that mean per-patient annual costs for DR care range from approximately INR 14,356 for non-sight-threatening DR to INR 31,820 for sight-threatening disease, with costs rising sharply with worsening visual impairment and need for interventional therapies [5]. In a healthcare system where out-of-pocket expenditure remains predominant, these costs frequently translate into catastrophic financial stress for affected households. Treatment intensity, monitoring requirements, and associated costs vary significantly across DR stages, making stage-specific economic analysis essential for clinical and health system planning. Catastrophic health expenditure (CHE), defined as out-of-pocket healthcare spending exceeding a substantial proportion of household capacity to pay, represents a critical indicator of financial risk in chronic disease. Treatment intensity and associated costs vary substantially across DR stages, making stage-specific analysis essential for clinical and policy planning

Beyond economic hardship, DR has a profound impact on vision-related quality of life (VRQoL). Patients with DR demonstrate significantly lower National Eye Institute 25-Item Visual Function Questionnaire (NEI-VFQ-25) composite scores compared with diabetics without retinopathy (mean 73.93 vs. 99.26), with the most severely affected domains being general health (58.43), general vision (63.69), and mental health [7,8]. VRQoL declines progressively with both the duration of DR (mean 10.98 years vs. 6.69 years in controls) and disease severity, showing strong negative correlations across nearly all functional subscales (all p<0.001) [9,10]. Mental-health subscales reflect persistent worry, frustration, and emotional distress related to visual decline, while near-vision activities and driving function are among the most severely compromised, emphasizing the broad functional consequences of DR in daily life.

Despite these well-recognized economic and quality-of-life consequences, existing Indian literature largely evaluates cost burden, visual disability, and psychosocial impact in isolation, often focuses only on advanced disease, and rarely provides prospective, grade-wise stratification across the full clinical spectrum of DR. Critically, there is a lack of integrated Indian data that simultaneously quantify direct and indirect costs, catastrophic health expenditure, patient coping strategies, and vision-related quality of life across all stages of diabetic retinopathy.

The present study was designed to evaluate stage-specific differences in economic burden, catastrophic health expenditure, household coping strategies, vision-related quality of life, and mental-health outcomes across the full clinical spectrum of diabetic retinopathy in an Indian tertiary-care setting, while also incorporating exploratory insurance projections to assess potential financial risk-protection strategies. Additionally, this study incorporates exploratory insurance projections to provide preliminary insights into potential financial risk-pooling strategies.

## Materials and methods

This cross-sectional observational study was conducted at a tertiary eye care center in Chennai, South India, between November 2024 and March 2025. The study protocol was approved by the Institutional Research Ethics Committee, Sri Ramachandra Institute of Higher Education and Research, Chennai (Approval No. CSP-MED/24/NOV/111/354, dated December 18, 2025), and the study adhered to the tenets of the Declaration of Helsinki. Written informed consent was obtained from all participants prior to enrolment.

Adult patients aged 30-75 years with established type 2 diabetes mellitus and a clinical diagnosis of diabetic retinopathy at any stage were eligible for inclusion. Participants were required to be willing to disclose healthcare-related financial information and able to complete study questionnaires independently or with assistance. Patients with other visually significant ocular comorbidities such as advanced glaucoma or age-related macular degeneration, those with cognitive impairment affecting reliable questionnaire responses, those with incomplete medical or financial records, and those unwilling to participate were excluded.

All participants underwent a comprehensive ophthalmic evaluation that included best-corrected visual acuity measurement using Snellen charts with subsequent conversion to logarithm of the minimum angle of resolution (logMAR) units for analysis, slit-lamp biomicroscopy, intraocular pressure measurement using Goldmann applanation tonometry, and dilated fundus examination by indirect ophthalmoscopy. Optical coherence tomography was performed to evaluate macular status in all cases, and fundus fluorescein angiography was carried out when clinically indicated. Diabetic retinopathy was classified according to the International Clinical Diabetic Retinopathy Disease Severity Scale [11] into mild non-proliferative diabetic retinopathy (NPDR; microaneurysms only), moderate NPDR (more than microaneurysms but less than severe non-proliferative disease), severe NPDR (defined by extensive intraretinal hemorrhages, venous beading, or prominent intraretinal microvascular abnormalities), and proliferative diabetic retinopathy (presence of retinal or optic disc neovascularization and/or vitreous or preretinal hemorrhage). In patients with asymmetric disease, classification was based on the more severely affected eye.

Economic assessment included the estimation of direct medical costs, direct non-medical costs, and indirect costs. Direct medical costs were collected using a structured questionnaire and verified from medical records and receipts whenever available. These costs included consultation fees, expenses for diagnostic investigations such as optical coherence tomography, fundus fluorescein angiography, and ultrasonography, expenditure on prescription medications for diabetic retinopathy and diabetes management, costs of laser photocoagulation, intravitreal injections of anti-vascular endothelial growth factor agents or corticosteroids, surgical interventions including vitrectomy and cataract surgery, and hospitalization and vision rehabilitation services when applicable. Direct non-medical costs were documented through patient interviews using a standardized cost diary and included transportation expenses for patients and accompanying persons, accommodation during hospital visits, costs of visual aids, special equipment or home modifications, and paid caregiving expenses where relevant. All costs were assessed for the 12-month period preceding the study visit.

Indirect costs were estimated using the human capital approach to quantify productivity losses attributable to visual impairment. These included income loss due to workdays missed for hospital visits and recovery (absenteeism), reduced productivity while at work due to visual symptoms (presenteeism), premature retirement or job modifications necessitated by visual disability, and income loss among family members providing caregiving support. Productivity losses were calculated using self-reported income, and for unemployed participants, minimum wage rates for Tamil Nadu were used as a proxy for opportunity cost. Short-term productivity losses were annualized, while long-term employment changes such as job modification or retirement were recorded but not fully monetized. Presenteeism was assessed using self-reported estimates of productivity reduction and was not based on a validated instrument.

Vision-related quality of life was assessed using the Indian Vision Function Questionnaire (IND-VFQ-33) [12], a validated instrument specifically developed for the Indian population. This questionnaire evaluates visual functioning across domains of general mobility and functioning, psychosocial impact, visual symptoms, distance vision, and reading and near-vision activities. Responses were transformed to a 0-100 scale, with higher scores indicating poorer vision-related quality of life (inverse scoring, in contrast to conventional instruments where higher scores indicate better quality of life). The questionnaire was administered by trained research personnel in the participant’s preferred language, including Tamil, Telugu, Hindi, or English. Validated translations of the Patient Health Questionnaire-9 (PHQ-9) and Generalized Anxiety Disorder-7 (GAD-7) were used, and assistance was provided to visually impaired participants when required [13,14].

Statistical analysis

The sample size was determined based on previous estimates of catastrophic health expenditure among Indian households, reported to be approximately 27% [15], with an absolute precision of 5% and a confidence level of 95%. This yielded a minimum required sample size of 303 participants, which was increased to 350 to account for a projected 15% rate of non-response or incomplete data. Catastrophic health expenditure (CHE) was defined as healthcare costs exceeding 40% of non-food household expenditure, in accordance with the World Health Organization methodology for estimating catastrophic health spending [16]. 

Continuous variables were summarized as mean with standard deviation or median with interquartile range, as appropriate, and categorical variables as frequencies and percentages. Distributional assumptions were tested using the Shapiro-Wilk test, and homogeneity of variances was tested using Levene’s test. Participants were stratified by diabetic retinopathy severity, and between-group comparisons were performed using one-way analysis of variance (ANOVA) for normally distributed continuous variables and the Kruskal-Wallis test for non-normally distributed variables. Categorical variables were analyzed using the Chi-square test, or Fisher’s exact test, where expected cell counts were less than five. For all variables showing a significant omnibus result, Bonferroni-corrected post-hoc pairwise comparisons were performed to identify which stage pairs differed; in tables, significant pairwise differences are indicated by superscript letters (a: mild NPDR vs. moderate NPDR; b: mild NPDR vs. severe NPDR; c: mild NPDR vs. PDR; d: moderate NPDR vs. severe NPDR; e: moderate NPDR vs. PDR; f: severe NPDR vs. PDR). F-statistics are reported with numerator and denominator degrees of freedom in the form F(df1, df2).

Multiple linear regression analyses were conducted to identify independent predictors of economic burden and vision-related quality of life after adjustment for age, sex, education level, income, duration of diabetes, HbA1c, health insurance status, and the presence of other diabetes-related complications. Multicollinearity was assessed using the variance inflation factor (VIF), with VIF < 5 indicating the absence of significant collinearity among predictors. Residuals were visually inspected for approximate normality using histograms and normal Q-Q plots, and for homoscedasticity using residuals-versus-fitted scatterplots. Cases with missing values on any model covariate were excluded from the regression analyses; complete-case analysis retained 340 of 350 participants (missingness 2.9%), and all reported coefficients refer to this analytical sample. The association between total economic burden and VRQoL score was examined using Spearman’s rank correlation coefficient, with the 95% confidence interval derived using Fisher’s z-transformation.

In addition, an exploratory, scenario-based break-even calculation was performed using stage-weighted mean cost estimates derived from the study cohort. Stage weighting used the observed cohort distribution (95 mild NPDR, 112 moderate NPDR, 78 severe NPDR, 65 PDR out of 350 participants). Annual premium requirements were estimated under a simplified hypothetical model assuming: (i) insurance enrolment at 40 years of age; (ii) DR-related costs accruing predominantly between ages 55 and 60; and (iii) no discounting, inflation adjustment, medical-cost-inflation correction, risk adjustment for individual-level factors (for example, glycemic control or blood pressure), or administrative loading for insurer costs, profit margins, or risk reserves. Because the present cohort demonstrated substantial rates of mild and moderate NPDR at ages below 55 years, costs accruing prior to age 55 are not captured in this scenario, and the resulting premium estimate is therefore likely to be conservative. These projections are illustrative only, are reported descriptively without inferential testing, and do not constitute formal actuarial estimates.

Statistical analysis was performed using IBM SPSS Statistics version 26.0 (IBM Corp., Armonk, NY, USA). A two-sided p-value of less than 0.05 was considered statistically significant for all analyses.

## Results

Demographic and clinical profile

A total of 350 patients with type 2 diabetes mellitus and diabetic retinopathy were included in the analysis. The demographic and clinical characteristics stratified by DR severity are summarized in Table 1.

**Table 1:** Demographic and clinical characteristics of study participants by DR severity. Values are presented as mean ± SD or n (%). Continuous variables satisfying assumptions of normality (Shapiro–Wilk p > 0.05) and variance homogeneity were compared using one-way ANOVA; categorical variables were compared using the Chi-square test, or Fisher’s exact test where expected cell counts were < 5. Superscript letters denote significant (Bonferroni-adjusted) pairwise differences: a, mild vs. moderate NPDR; b, mild vs. severe NPDR; c, mild NPDR vs. PDR; d, moderate vs. severe NPDR; e, moderate NPDR vs. PDR; f, severe NPDR vs. PDR. DR, diabetic retinopathy; NPDR, non-proliferative diabetic retinopathy; PDR, proliferative diabetic retinopathy; INR, Indian National Rupee; HbA1c, glycated hemoglobin; logMAR, logarithm of the minimum angle of resolution.

Characteristic	Mild NPDR (n=95)	Moderate NPDR (n=112)	Severe NPDR (n=78)	PDR (n=65)	Test statistic	p-value
Age (years), mean ± SD	54.3 ± 8.2^b,c^	56.7 ± 7.8^e^	58.4 ± 8.5^a^	60.2 ± 7.3^a,b^	F(3, 346) = 7.95	0.002
Male, n (%)	52 (54.7)	65 (58.0)	45 (57.7)	40 (61.5)	χ² = 0.74	0.864
Female, n (%)	43 (45.3)	47 (42.0)	33 (42.3)	25 (38.5)		
No formal education, n (%)	17 (17.9)	22 (19.6)	20 (25.6)	18 (27.7)	χ² = 6.02	0.041
Primary school, n (%)	32 (33.7)	40 (35.7)	30 (38.5)	25 (38.5)		
Secondary school, n (%)	33 (34.7)	35 (31.3)	20 (25.6)	17 (26.2)		
College or higher, n (%)	13 (13.7)	15 (13.4)	8 (10.3)	5 (7.7)		
<15,000 INR, n (%)	21 (22.1)	30 (26.8)	25 (32.1)	24 (36.9)	χ² = 7.54	0.032
15,000–30,000 INR, n (%)	42 (44.2)	50 (44.6)	35 (44.9)	28 (43.1)		
30,001–50,000 INR, n (%)	22 (23.2)	20 (17.9)	12 (15.4)	10 (15.4)		
>50,000 INR, n (%)	10 (10.5)	12 (10.7)	6 (7.7)	3 (4.6)		
Health insurance coverage, n (%)	18 (18.9)	22 (19.6)	12 (15.4)	9 (13.8)	χ² = 8.05	0.045
Diabetes duration (years), mean ± SD	7.2 ± 3.8^a,b,c^	10.5 ± 4.2^a,d,e^	13.8 ± 5.1^b,d,f^	15.7 ± 4.9^c,e,f^	F(3, 346) = 57.45	<0.001
HbA1c (%), mean ± SD	7.8 ± 1.2^a,b,c^	8.3 ± 1.4^a,d,e^	9.1 ± 1.6^b,d,f^	9.8 ± 1.8^c,e,f^	F(3, 346) = 27.99	<0.001
Nephropathy, n (%)	12 (12.6)	23 (20.5)	27 (34.6)	31 (47.7)	χ² = 29.00	<0.001
Neuropathy, n (%)	25 (26.3)	38 (33.9)	35 (44.9)	38 (58.5)	χ² = 19.14	<0.001
Cardiovascular disease, n (%)	18 (18.9)	30 (26.8)	28 (35.9)	30 (46.2)	χ² = 15.35	<0.001
Visual acuity (logMAR), mean ± SD	0.22 ± 0.14^a,b,c^	0.38 ± 0.22^a,d,e^	0.67 ± 0.31^b,d,f^	0.95 ± 0.42^c,e,f^	F(3, 346) = 108.90	<0.001
Diabetic macular edema, n (%)	8 (8.4)	25 (22.3)	32 (41.0)	35 (53.8)	χ² = 47.32	<0.001

The mean age of participants increased progressively with disease severity, from 54.3 ± 8.2 years in mild NPDR to 60.2 ± 7.3 years in PDR (p = 0.002); post-hoc testing showed that mild NPDR differed significantly from both severe NPDR and PDR, and moderate NPDR differed significantly from PDR. Male patients constituted a slight majority across all stages, with no significant gender difference between groups.

Educational attainment and household income were inversely associated with DR severity. The proportion of patients without formal education increased from 17.9% in mild NPDR to 27.7% in PDR, while the proportion earning more than INR 50,000 per month declined from 10.5% to 4.6% across the same spectrum (p = 0.041 and p = 0.032, respectively). Health insurance coverage was uniformly low and showed a modest decrease with increasing DR severity, from 18.9% in mild NPDR to 13.8% in PDR (p = 0.045).

Clinical severity markers demonstrated strong stepwise progression across the DR spectrum. Mean duration of diabetes increased from 7.2 ± 3.8 years in mild NPDR to 15.7 ± 4.9 years in PDR (p < 0.001), while mean HbA1c levels rose from 7.8 ± 1.2% to 9.8 ± 1.8% (p < 0.001); for both variables, Bonferroni-adjusted post-hoc comparisons showed that all six between-stage pairwise differences were statistically significant. The prevalence of systemic diabetic complications increased significantly with DR severity, including nephropathy (12.6% to 47.7%), neuropathy (26.3% to 58.5%), and cardiovascular disease (18.9% to 46.2%) (all p < 0.001).

Visual function deteriorated markedly with advancing retinopathy. Mean best-corrected visual acuity worsened from 0.22 ± 0.14 logMAR in mild NPDR to 0.95 ± 0.42 logMAR in PDR (p < 0.001), with all pairwise stage comparisons remaining significant after Bonferroni correction. The prevalence of diabetic macular edema increased nearly seven-fold from mild NPDR to PDR (8.4% vs. 53.8%, p < 0.001), highlighting the sharp escalation in macular involvement with disease progression.

Economic burden across DR severity

A pronounced and statistically significant escalation in economic burden was observed with increasing DR severity (Table 2). Mean total direct medical costs increased more than six-fold across the disease spectrum, from INR 15,000 ± 2,500 in mild NPDR to INR 95,000 ± 12,000 in PDR (p < 0.001), with all between-stage pairwise differences remaining significant after Bonferroni correction. Among direct medical components, expenditures on diagnostic investigations, laser therapy, and pharmacologic treatment increased progressively with disease severity. Intravitreal anti-vascular endothelial growth factor (anti-VEGF) therapy emerged as a major driver of direct medical expenditure in severe NPDR and PDR, contributing approximately one-third of total direct medical costs in these groups, although diagnostic investigations and pharmacologic therapy collectively exceeded anti-VEGF costs at every stage.

**Table 2:** Direct medical costs, indirect costs, total economic burden, and catastrophic health expenditure by DR severity. Cost values are expressed as mean ± SD (INR) unless otherwise stated. Catastrophic health expenditure is expressed as n (%) of participants within each stage whose out-of-pocket healthcare spending exceeded 40% of non-food household expenditure. Economic burden as % of annual household income is expressed as mean ± SD. Between-stage comparisons used one-way ANOVA for continuous variables and the Chi-square test for categorical variables. Superscript letters denote significant (Bonferroni-adjusted) pairwise differences: a, mild vs. moderate NPDR; b, mild vs. severe NPDR; c, mild NPDR vs. PDR; d, moderate vs. severe NPDR; e, moderate NPDR vs. PDR; f, severe NPDR vs. PDR. 95% CIs are shown for total economic burden. DR, diabetic retinopathy; NPDR, non-proliferative diabetic retinopathy; PDR, proliferative diabetic retinopathy; VEGF, vascular endothelial growth factor; INR, Indian National Rupee.

Cost category	Mild NPDR	Moderate NPDR	Severe NPDR	PDR	Test statistic	p-value
Consultations	2,500 ± 800^a,b,c^	4,800 ± 1,200^a,d,e^	8,000 ± 1,800^b,d,f^	10,500 ± 2,200^c,e,f^	F(3, 346) = 436.96	<0.001
Diagnostic tests	3,800 ± 1,200^a,b,c^	7,500 ± 1,800^a,d,e^	12,000 ± 2,500^b,d,f^	15,500 ± 3,000^c,e,f^	F(3, 346) = 465.79	<0.001
Medications	6,200 ± 1,500^a,b,c^	9,700 ± 2,200^a,d,e^	14,000 ± 2,800^b,d,f^	18,000 ± 3,500^c,e,f^	F(3, 346) = 337.04	<0.001
Laser procedures	2,500 ± 1,000^a,b,c^	8,000 ± 2,000^a,d,e^	15,000 ± 3,000^b,d,f^	18,000 ± 3,800^c,e,f^	F(3, 346) = 636.81	<0.001
Anti-VEGF injections	0	5,000 ± 3,500^a,d,e^	23,000 ± 7,000^b,d,f^	30,000 ± 8,500^c,e,f^	F(3, 346) = 586.73	<0.001
Surgical interventions	0	0	0	3,000 ± 8,000^c,e,f^	F(3, 346) = 13.41	<0.001
Total direct medical cost	15,000 ± 2,500^a,b,c^	35,000 ± 4,800^a,d,e^	72,000 ± 8,500^b,d,f^	95,000 ± 12,000^c,e,f^	F(3, 346) = 1998.39	<0.001
Transportation	1,800 ± 500^a,b,c^	4,200 ± 1,000^a,d,e^	8,500 ± 1,800^b,d,f^	12,000 ± 2,500^c,e,f^	F(3, 346) = 715.77	<0.001
Accommodation	200 ± 300^a,b,c^	800 ± 600^a,d,e^	2,500 ± 1,000^b,d,f^	5,000 ± 1,500^c,e,f^	F(3, 346) = 409.69	<0.001
Lost productivity (patient)	2,500 ± 800^a,b,c^	5,000 ± 1,500^a,d,e^	10,000 ± 2,500^b,d,f^	12,000 ± 3,000^c,e,f^	F(3, 346) = 385.82	<0.001
Lost productivity (caregiver)	500 ± 300^a,b,c^	2,000 ± 800^a,d,e^	4,000 ± 1,200^b,d,f^	6,000 ± 1,800^c,e,f^	F(3, 346) = 376.53	<0.001
Total indirect costs	5,000 ± 1,200^a,b,c^	12,000 ± 2,500^a,d,e^	25,000 ± 4,000^b,d,f^	35,000 ± 6,000^c,e,f^	F(3, 346) = 627.09	<0.001
Total economic burden (INR)	20,000 ± 3,000^a,b,c^ (95% CI 19,396–20,604)	47,000 ± 5,000^a,d,e^ (95% CI 46,074-47,926)	97,000 ± 9,000^b,d,f^ (95% CI 95,003-98,997)	130,000 ± 13,000^c,e,f^ (95% CI 126,840-133,160)	F(3, 346) = 1738.34	<0.001
Economic burden as % of annual household income	8.2 ± 3.1^a,b,c^	18.5 ± 5.4^a,d,e^	35.2 ± 8.6^b,d,f^	48.6 ± 10.3^c,e,f^	F(3, 346) = 412.87	<0.001
Catastrophic health expenditure, n (%)	11 (12)	31 (28)	51 (65)	51 (78)	χ² = 135.4	<0.001

Indirect costs also showed a steep severity-dependent gradient. Mean total indirect costs increased from INR 5,000 ± 1,200 in mild NPDR to INR 35,000 ± 6,000 in PDR (p < 0.001). Lost productivity among patients constituted the largest proportion of indirect costs, followed by caregiver productivity losses, with both components increasing significantly with worsening DR stage.

When combined, the mean total economic burden (direct + indirect) rose more than sixfold across the disease spectrum, from INR 20,000 ± 3,000 (95% CI 19,396-20,604) in mild NPDR to INR 130,000 ± 13,000 (95% CI 126,840-133,160) in PDR (p < 0.001). Expressed as a proportion of annual household income, the economic burden increased from 8.2% ± 3.1% in mild NPDR to a catastrophic 48.6% ± 10.3% in PDR (p < 0.001). This represents a transition from manageable expenditure in early disease to catastrophic financial burden in advanced stages. The relative contribution of indirect costs to total expenditure increased modestly with disease severity, underscoring the growing impact of productivity losses in advanced DR.

Vision-related quality of life and mental health

Vision-related quality of life deteriorated progressively and significantly across all domains with increasing DR severity (Table 3). The overall VRQoL score worsened from 30 ± 8 in mild NPDR to 90 ± 10 in PDR (p < 0.001), with Bonferroni-adjusted post-hoc testing confirming significant differences for all six between-stage pairs. The most severely affected functional domains in advanced disease were reading and near-vision activities (95 ± 8) and distance vision (92 ± 9), reflecting profound impairment in daily visual tasks.

**Table 3:** Vision-related quality of life and mental health scores by DR severity. Values are mean ± SD. Higher VRQoL scores indicate poorer vision-related quality of life (inverse scoring). Between-stage comparisons used one-way ANOVA. Superscript letters denote significant (Bonferroni-adjusted) pairwise differences: a, mild vs. moderate NPDR; b, mild vs. severe NPDR; c, mild NPDR vs. PDR; d, moderate vs. severe NPDR; e, moderate NPDR vs. PDR; f, severe NPDR vs. PDR. DR, diabetic retinopathy; GAD-7, Generalized Anxiety Disorder-7; NPDR, non-proliferative diabetic retinopathy; PHQ-9, Patient Health Questionnaire-9; VRQoL, vision-related quality of life.

VRQoL domain/mental health	Mild NPDR	Moderate NPDR	Severe NPDR	PDR	Test statistic	p-value
General functioning/mobility	22 ± 8^a,b,c^	42 ± 12^a,d,e^	68 ± 15^b,d,f^	85 ± 10^c,e,f^	F(3, 346) = 405.68	<0.001
Psychosocial impact	25 ± 10^a,b,c^	48 ± 14^a,d,e^	70 ± 16^b,d,f^	88 ± 12^c,e,f^	F(3, 346) = 344.77	<0.001
Visual symptoms	30 ± 12^a,b,c^	52 ± 15^a,d,e^	72 ± 14^b,d,f^	86 ± 11^c,e,f^	F(3, 346) = 301.72	<0.001
Color vision	18 ± 9^a,b,c^	35 ± 12^a,d,e^	55 ± 16^b,d,f^	70 ± 15^c,e,f^	F(3, 346) = 183.88	<0.001
Distance vision	28 ± 11^a,b,c^	50 ± 14^a,d,e^	75 ± 15^b,d,f^	92 ± 9^c,e,f^	F(3, 346) = 420.44	<0.001
Reading/near vision	35 ± 12^a,b,c^	65 ± 15^a,d,e^	85 ± 12^b,d,f^	95 ± 8^c,e,f^	F(3, 346) = 363.72	<0.001
Overall VRQoL score	30 ± 8^a,b,c^	55 ± 12^a,d,e^	75 ± 15^b,d,f^	90 ± 10^c,e,f^	F(3, 346) = 444.66	<0.001
Depression (PHQ-9)	4.2 ± 2.5^a,b,c^	7.8 ± 3.5^a,d,e^	12.5 ± 4.2^b,d,f^	15.8 ± 4.5^c,e,f^	F(3, 346) = 201.49	<0.001
Anxiety (GAD-7)	3.8 ± 2.2^a,b,c^	6.5 ± 3.0^a,d,e^	10.8 ± 3.8^b,d,f^	14.5 ± 4.2^c,e,f^	F(3, 346) = 258.36	<0.001

Psychosocial impact also worsened markedly with disease progression, with mean domain scores increasing from 25 ± 10 in mild NPDR to 88 ± 12 in PDR (p < 0.001). Mental health burden mirrored these functional declines. Mean PHQ-9 depression scores increased from 4.2 ± 2.5 in mild NPDR to 15.8 ± 4.5 in PDR, while mean GAD-7 anxiety scores rose from 3.8 ± 2.2 to 14.5 ± 4.2 across the same spectrum (both p < 0.001). Notably, the mean PHQ-9 score in PDR patients (15.8) exceeded the threshold for severe depression (≥ 15), indicating that the average patient with advanced DR experiences clinically significant depressive symptoms. Severe anxiety and/or depression (score ≥ 15) was reported by approximately 15% of patients with PDR, compared with only 2% of those with mild NPDR.

Association between economic burden and quality of life

**Figure 1:**
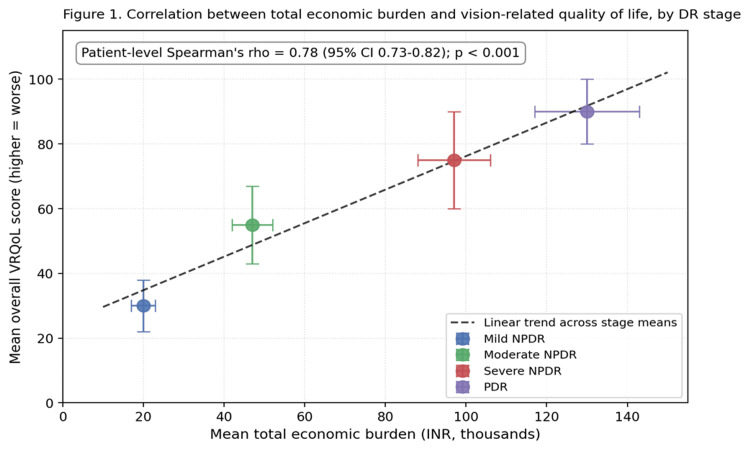
Correlation between total economic burden and overall vision-related quality of life, shown by DR stage (mean ± SD). DR, diabetic retinopathy; VRQoL, vision-related quality of life (higher = worse); INR, Indian National Rupee; NPDR, non-proliferative diabetic retinopathy.

A strong positive correlation was observed between total economic burden and overall VRQoL score (Spearman’s ρ = 0.78, 95% CI 0.73-0.82; p < 0.001), indicating that higher financial burden was closely associated with poorer vision-related quality of life (Figure 1).

On multivariable regression analysis, both DR severity and total economic burden remained independent predictors of worse VRQoL after adjustment for age, sex, education, income, duration of diabetes, HbA1c, and the presence of other systemic complications (Table 4). Higher DR stage, longer duration of diabetes, poorer glycemic control, lower educational attainment, and lack of health insurance were all significantly associated with worse VRQoL. Each INR 10,000 increase in total economic burden was independently associated with a 1.8-point worsening in overall VRQoL score (95% CI 1.2-2.4; p < 0.001), independent of DR stage and other covariates. All VIF values were below 2.4, indicating the absence of significant multicollinearity, and inspection of residuals showed no material departure from normality or homoscedasticity.

**Table 4:** Multiple linear regression analysis of factors associated with vision-related quality of life. β coefficients represent the change in overall VRQoL score (higher = worse) per unit increase in the predictor. Reference categories: mild NPDR (for DR severity); male (for gender); no formal education (for education); < 15,000 INR per month (for income); and no health insurance coverage (for insurance). All VIF values were < 2.4 (no significant multicollinearity); residuals were approximately normally distributed with homogeneous variance. Complete-case analysis (n = 340). DR, diabetic retinopathy; HbA1c, glycated hemoglobin; INR, Indian National Rupee; NPDR, non-proliferative diabetic retinopathy; VIF, variance inflation factor; VRQoL, vision-related quality of life.

Variable	Unstandardized β	95% CI	Standardized β	p-value
Moderate NPDR	15.6	10.2–21.0	0.31	<0.001
Severe NPDR	28.4	22.5–34.3	0.52	<0.001
PDR	35.8	29.4–42.2	0.61	<0.001
Economic burden (per 10,000 INR)	1.8	1.2–2.4	0.25	<0.001
Age (per 10 years)	2.1	0.8–3.4	0.09	0.002
Female gender	3.5	0.9–6.1	0.07	0.008
Secondary education	-4.6	-8.0 to -1.2	-0.09	0.008
College or higher	-7.8	-12.3 to -3.3	-0.11	0.001
Income 30,001–50,000	-5.8	-9.9 to -1.7	-0.09	0.006
Income >50,000	-8.5	-13.8 to -3.2	-0.10	0.002
Diabetes duration	2.3	1.5–3.1	0.18	<0.001
HbA1c	1.9	1.1–2.7	0.12	<0.001
Other complications	4.2	1.6–6.8	0.08	0.002
Health insurance	-6.5	-9.8 to -3.2	-0.10	<0.001

Catastrophic health expenditure and coping strategies

Overall, 42% of the study population experienced catastrophic health expenditure, defined as healthcare spending exceeding 40% of non-food household expenditure. The prevalence of catastrophic expenditure increased sharply from mild NPDR to PDR, affecting 12% of patients with mild NPDR, 28% with moderate NPDR, 65% with severe NPDR, and 78% with PDR (p < 0.001); these values are also included in Table 2.

**Figure 2:**
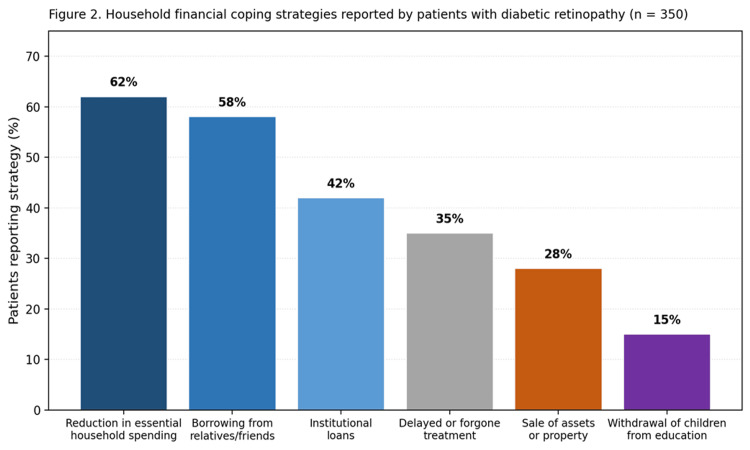
Overall distribution of household financial coping strategies among 350 patients with diabetic retinopathy.

Patients reported multiple coping strategies to manage the financial burden of care (Figure 2).

The most common strategies included reduction of essential household expenditure (62%), borrowing money from relatives or friends (58%), taking institutional loans (42%), and selling assets or property (28%). Social consequences were substantial, with 15% of households reporting withdrawal of children from education due to financial strain. Treatment compromise was common, with 35% of patients overall and 68% of patients with PDR reporting delayed or forgone recommended treatment because of cost constraints.

Exploratory insurance premium projections

Using stage-weighted mean cost estimates derived from the observed distribution of disease severity in the study cohort (95 mild NPDR, 112 moderate NPDR, 78 severe NPDR, and 65 PDR participants out of 350), an exploratory insurance projection was performed to estimate the annual premium requirement under a simplified life-course scenario. Using stage-weighted estimates derived from the mean values reported in Table 2 and the observed cohort distribution, the mean annual direct medical cost of diabetic retinopathy across the cohort was approximately INR 49,000 per patient, while the corresponding total economic burden (direct + indirect) was approximately INR 66,000 per patient per year.

Under a hypothetical model assuming insurance enrolment at 40 years of age, with diabetic retinopathy-related costs predominantly accruing during the 55-60 year age window, the cumulative 5-year expected cost for direct medical care was estimated at approximately INR 2.45 lakh per patient, while the cumulative total economic burden was estimated at approximately INR 3.30 lakh per patient. When these costs were amortized over a 15-year contribution period (ages 40-55), the corresponding minimum annual insurance premium requirement was estimated at approximately INR 16,000 per year to cover direct medical costs alone and approximately INR 22,000 per year to cover the full economic burden. As detailed in the Methods, this projection does not account for discounting, inflation, medical-cost inflation, individual-level risk adjustment, administrative loading, or costs arising prior to age 55, and real-world premium requirements would therefore be higher than the figures reported above.

## Discussion

This prospective, stage-stratified analysis demonstrates that diabetic retinopathy in India is associated with a steep and progressive escalation in economic burden, functional disability, and psychosocial distress across the full clinical spectrum from mild non-proliferative disease to proliferative diabetic retinopathy. We show a more than six-fold rise in total annual per-patient costs between early and advanced stages of DR, with anti-VEGF therapy emerging as the dominant driver of direct medical expenditure and productivity losses constituting an increasingly large proportion of total costs. Importantly, nearly half of patients with proliferative disease experienced catastrophic health expenditure, and a substantial proportion resorted to distress-financing strategies such as borrowing, asset liquidation, reduction in essential household spending, and treatment deferral. Vision-related quality of life deteriorated sharply with advancing DR severity across all functional domains, accompanied by a parallel rise in clinically significant symptoms of depression and anxiety. Finally, our exploratory insurance projections suggest that relatively modest, stage-weighted annual contributions initiated in mid-life may help offset part of the catastrophic financial burden associated with advanced diabetic retinopathy, although these projections should be interpreted cautiously given the simplified assumptions underlying the model. Together, these findings position diabetic retinopathy as not only a vision-threatening complication but also a major contributor to severe financial distress and psychosocial vulnerability during the most economically productive years of life.

The progressive increase in healthcare expenditure with worsening severity of diabetic retinopathy is not unexpected and has been consistently documented across diverse healthcare systems, particularly in low- and middle-income countries (LMICs), where out-of-pocket payment remains the dominant mode of financing [5,6]. Recent studies have further confirmed a marked stage-dependent rise in healthcare utilization and treatment-related costs as diabetic retinopathy progresses from non-proliferative to proliferative stages [17-20]. In such settings, the cost of advanced diagnostics, repeated intravitreal therapies, laser procedures, and vitreoretinal surgery predictably escalates as disease severity increases [5,21]. The substantial contribution of anti-VEGF therapy to overall retinal disease expenditure observed in our cohort is also consistent with previous reports demonstrating that repeated intravitreal injections constitute a major component of long-term treatment burden in retinal vascular diseases, particularly in healthcare systems where out-of-pocket expenditure remains predominant. Indian studies evaluating adherence to intravitreal anti-VEGF therapy have similarly highlighted the significant financial burden associated with repeated injections and long-term treatment follow-up requirements in patients with diabetic macular edema and other retinal vascular disorders [22]. Our findings, therefore, align with the broader global and Indian literature in demonstrating a steep upward cost gradient with advancing DR. However, what has remained inadequately characterized in existing studies is the magnitude of this escalation at each distinct stage of DR, and its translation into household-level financial catastrophe, which our stage-stratified analysis was specifically designed to address. The progressive worsening of glycemic control observed across DR stages in our cohort is also important in interpreting the observed cost gradients. Mean HbA1c levels increased significantly from mild NPDR to proliferative disease, suggesting that poor metabolic control may contribute both to disease progression and to increasing treatment intensity and healthcare expenditure. Importantly, multivariable regression analyses adjusted for HbA1c levels, indicating that the observed associations between DR severity, economic burden, and worsening vision-related quality of life remained significant independent of glycemic control.

A key contribution of the present study is the robust quantification of catastrophic health expenditure across the full DR severity spectrum, with more than three-quarters of patients with proliferative disease experiencing healthcare spending that exceeded 40% of non-food household expenditure. National and state-level analyses of non-communicable diseases in India and abroad have demonstrated substantial levels of catastrophic healthcare expenditure among households affected by chronic conditions [15,17,23,24], including diabetes, highlighting the financial fragility associated with long-term disease management. While previous Indian studies have acknowledged high out-of-pocket expenditure for diabetes and its complications [3,15], formal, definition-based reporting of catastrophic expenditure specific to DR remains sparse. By demonstrating a sharp inflexion in catastrophic spending from severe NPDR onward, our data identify the precise clinical stage at which households transition from financial strain to economic destabilization.

Beyond financial-risk protection strategies, scalable low-cost service delivery models may also play an important role in reducing the long-term economic burden of diabetic retinopathy. Tele-ophthalmology screening programs, artificial intelligence-assisted retinal image analysis, and task-sharing approaches involving trained optometrists and vision technicians may facilitate earlier detection of retinopathy in resource-constrained settings. Earlier diagnosis and timely intervention may reduce progression to advanced disease stages requiring costly anti-VEGF therapy, repeated laser procedures, and vitreoretinal surgery, thereby potentially decreasing catastrophic health expenditure at the population level. In addition, decentralized community-based screening programs may improve accessibility for rural and underserved populations who frequently present late with advanced disease due to limited access to specialist retinal care. Integration of AI-assisted DR screening into primary diabetes clinics may further reduce referral delays and improve cost-efficiency by prioritizing high-risk patients requiring specialist evaluation. Such approaches may be particularly relevant in low- and middle-income settings like India, where the growing burden of diabetes substantially exceeds the capacity of existing tertiary retinal care services. Over time, these preventive and early-intervention models may reduce both healthcare-system expenditure and indirect productivity losses associated with vision impairment and visual disability.

Perhaps the most disturbing, and least previously documented, finding of this study pertains to family-level coping strategies adopted in response to DR-related financial stress. Beyond borrowing, loans, and asset liquidation, a striking 15% of households reported withdrawal of children from formal education to offset treatment expenses. This observation suggests that in LMIC settings, the consequences of advanced diabetic retinopathy may extend beyond the affected individual and raise concerns regarding potential intergenerational socioeconomic consequences that warrant further investigation. To our knowledge, educational disruption related to DR-associated healthcare expenditure has been insufficiently explored in ophthalmic literature and warrants further longitudinal investigation.

While prevention through sustained glycemic control and early retinopathy screening remains the cornerstone of reducing both clinical and economic burden, our findings suggest that prevention alone is unlikely to be sufficient in the absence of parallel financial-risk protection mechanisms. In this context, the exploratory insurance projections generated from our cohort data represent a novel translational component of this study. By illustrating that stage-weighted annual contributions initiated earlier in life might potentially help offset part of the later catastrophic costs associated with advanced diabetic retinopathy, our model offers a preliminary conceptual illustration of how disease prevention strategies and financial-risk protection mechanisms might potentially be aligned. At an annual premium of approximately INR 22,000, the projected insurance contribution would correspond to roughly 6-8% of annual household income for a typical middle-income Indian family, and up to 9-10% in lower-income households, underscoring the need for targeted subsidies to ensure equitable coverage. Rather than developing entirely disease-specific insurance products, integration of diabetic retinopathy screening and treatment pathways into existing publicly funded healthcare financing systems may represent a more feasible and equitable strategy in the Indian context. Existing schemes such as Ayushman Bharat Pradhan Mantri Jan Arogya Yojana (PM-JAY), the Tamil Nadu Chief Minister’s Comprehensive Health Insurance Scheme, and Employee State Insurance Corporation (ESIC)-based healthcare coverage may provide potential platforms for expanding financial protection for diabetic retinopathy-related care. Strengthening coverage for retinal screening, intravitreal therapy, laser procedures, and vitreoretinal surgery within such schemes may reduce out-of-pocket expenditure and improve timely access to care for economically vulnerable populations.

Importantly, while a stage-linked or incentive-based insurance structure could theoretically encourage improved glycemic control and regular ophthalmic screening, such models also raise important ethical and equity-related concerns. Individuals with poor glycemic control or advanced diabetic retinopathy are often those facing greater socioeconomic barriers to healthcare access, delayed diagnosis, and reduced treatment adherence. Consequently, linking insurance premiums to disease severity may unintentionally penalize vulnerable populations and potentially exacerbate existing healthcare inequities. Additional practical concerns include methods for verification of disease stage, frequency of reassessment, affordability for individuals diagnosed at advanced stages of disease, and regulatory feasibility within Indian insurance frameworks. Accordingly, the present proposal should be interpreted strictly as a preliminary conceptual illustration rather than a recommendation for direct implementation.

At the same time, implementation of any health-stage-linked or incentive-based insurance framework raises important concerns regarding data privacy, ethical governance, and responsible use of personal medical information. Any linkage between retinal disease severity, glycemic control, or treatment adherence and insurance pricing carries potential risks related to discriminatory underwriting practices, exclusion of high-risk individuals, and widening of existing healthcare inequities. These concerns may be particularly relevant in low- and middle-income settings, where socioeconomic barriers already influence access to diabetic care and long-term treatment adherence. Consequently, any future integration of clinical ophthalmic data into insurance-related decision-making would require robust ethical safeguards, transparent consent processes, and strict limitations on secondary use of patient health data. Such frameworks would also need to operate in compliance with India’s Digital Personal Data Protection Act, 2023, which emphasizes lawful processing of personal health information, informed consent, data minimization, and accountability in handling sensitive digital health records. In addition, strong regulatory oversight would be essential to ensure that financial-risk protection mechanisms remain equitable, inclusive, and accessible to vulnerable patient populations rather than functioning in a discriminatory or exclusionary manner

The findings of this study also have important implications for clinical practice. Given the high prevalence of catastrophic expenditure, depressive symptoms, and treatment deferral in advanced disease, ophthalmologists managing diabetic retinopathy may need to incorporate screening for financial distress and psychosocial burden into routine care.

The major strengths of this study include its prospective design, comprehensive stage-wise stratification across the full spectrum of diabetic retinopathy, and the integrated assessment of direct and indirect costs, catastrophic health expenditure, vision-related quality of life, and mental-health outcomes within a single analytical framework. This study systematically documents real-world household coping strategies related to diabetic retinopathy-associated financial stress while also proposing an exploratory insurance framework-elements that, to our knowledge, have not previously been examined together in Indian diabetic retinopathy research. Nevertheless, the study has certain limitations. Being conducted at a single tertiary-care referral center, the findings may over-represent advanced disease and may not be fully generalizable to community-based populations. Cost estimates were partly based on self-reported expenditure and income and are therefore subject to recall bias. The insurance projections are scenario-based and illustrative rather than actuarial estimates. Finally, the cross-sectional nature of the analysis precludes causal inference between economic burden, quality of life, and psychosocial outcomes.

Limitations

Several limitations should be considered when interpreting these findings. First, being conducted at a single tertiary-care referral center, the study likely over-represented patients with advanced disease, higher treatment intensity, and greater healthcare utilization compared with community-based diabetic populations. Consequently, the reported economic burden estimates may represent upper-bound estimates relative to the broader population of patients with diabetic retinopathy in India and may not be fully generalizable to non-tertiary care settings. Second, cost estimates relied partly on self-reported expenditure and income data, introducing the possibility of recall bias, although verification against available medical records and receipts was performed where feasible. Third, presenteeism estimates were based on patient self-report and were not assessed using a validated productivity-loss instrument. Fourth, indirect costs were estimated using the human capital approach, which may overestimate productivity losses in settings with variable employment patterns and underemployment. Fifth, no adjustment was made for multiple comparisons across all exploratory analyses, increasing the possibility of type I error. Sixth, health insurance status was self-reported and not independently verified against policy documents. Seventh, the study did not assess reasons underlying the lack of insurance coverage, including affordability, awareness, or accessibility barriers. Eighth, the exploratory insurance projections were illustrative rather than actuarial estimates and did not incorporate discounting, inflation adjustment, medical-cost inflation, administrative loading, or costs incurred before 55 years of age. Finally, findings from a tertiary-care center in Chennai may not be generalizable to other regions of India with different healthcare infrastructures and economic conditions.

These findings underscore the need for integrated clinical, economic, and financial risk-protection strategies in the management of diabetic retinopathy in India. Future research should incorporate longitudinal designs and actuarially robust modeling approaches to refine cost projections and inform scalable insurance frameworks.

## Conclusions

Diabetic retinopathy in India imposes a steep, stage-dependent escalation in economic burden, catastrophic health expenditure, and deterioration in vision-related quality of life, with profound psychosocial and intergenerational consequences. Our findings highlight that financial toxicity becomes particularly severe from advanced non-proliferative stages onward. The exploratory insurance projections provide a preliminary conceptual illustration of how risk-pooling approaches initiated earlier in life might potentially help mitigate later catastrophic healthcare expenditure associated with advanced diabetic retinopathy. Alongside prevention through glycemic control and early screening, integrated financial-risk protection is essential for sustainable DR care in low- and middle-income settings.
